# Isolation and purification of lipoarabinomannan from urine of adults with active TB

**DOI:** 10.5588/ijtld.22.0372

**Published:** 2023-01-01

**Authors:** J. L. Cantera, A. A. Rashid, L. M. Lillis, R. B. Peck, P. K. Drain, A. E. Shapiro, D. P. K. Wilson, A. Pinter, M. Kawasaki, E. Moreau, D. S. Boyle

**Affiliations:** 1 PATH, Seattle, WA,; 2 Department of Global Health and Medicine, University of Washington, Seattle, WA, USA; 3 Department of Epidemiology, University of Washington, Seattle, WA, USA; 4 Umkhuseli Research and Innovation Management and University of Kwa-Zulu Natal, Pietermaritzburg, South Africa; 5 Public Health Research Institute, New Jersey Medical School, Rutgers University, Newark, NJ, USA; 6 Otsuka Pharmaceutical, Tokyo, Japan; 7 FIND, Geneva, Switzerland; 8 Quotient Limited, Eysins, Switzerland

Dear Editor,

We report a multi-step approach to purify lipoarabinomannan (LAM) from urine from patients confirmed with active TB. LAM is a lipoglycan and a virulence factor associated with *Mycobacterium tuberculosis* (MTB). It has a glycerophospholipid terminus that non-covalently anchors the molecule within the inner cell membrane and attaches to a common mannan domain.^1^ The presence and number of sugars that cap the terminal residue of the arabinan side chains can be used to discriminate different species of mycobacteria.^2^ Soluble LAM is relatively abundant, as it is produced in both pulmonary and extrapulmonary TB disease, and actively secreted in exosomes from bacteria and infected macrophages; it is excreted in urine, making it a compelling biomarker for non-invasive rapid diagnostic tests (RDTs) for TB.^1^ Several RDTs targeting urinary LAM (uLAM) have been described,^3^,^4^ but none are sensitive enough to reliably detect uLAM for most cases of active TB. The limited diagnostic performance of LAM immunoassays has been due in part to employing LAM antibodies that were generated and/or screened using either in vitro cultured MTB cells, immunogens purified from in vitro cultured cells, and/or using culture-derived LAM (cLAM),^5^–^9^ rather than LAM from in vivo clinical specimens. While this is convenient in terms of producing sufficient quantity and high-quality material for antibody development, in vivo grown MTB may produce LAM with significant structural differences to cLAM.^10^,^11^ These structural differences may have a profound impact and compromise the clinical performance of LAM RDTs if the anti-LAM antibodies do not recognize the key epitopes found only in uLAM derivatives. As such, uLAM is critical for developing and/or assessing novel uLAM-specific antibodies for use in LAM-based RDTs.

To demonstrate scaled extraction of uLAM, a 500-mL contrived urine was prepared by pooling aliquots from 40 TB-positive patients (FIND, Geneva, Switzerland) into 460-mL urine from healthy individuals (BioIVT, NY, USA). Written informed consent, study protocols, and collection details for TB-positive patients have been described previously.^12^ For each sequential step, uLAM was measured using a biplexed sandwich immunoassay with electrochemiluminescent detection.^13^ The contrived urine was subjected to low-speed centrifugation to remove visible precipitate formed after prolonged freezer storage. LAM was not detected in the pellet from this initial centrifugation (Supplementary Figure S1). The supernatant was passed through a 0.2-μm filter (Sarstedt, NV, USA) to further remove small particulates (Step 1). The total uLAM in the filtrate was about 486.9 ng or 102.8 ng using FIND28/A194 (FA) and S4-20/A194 (OA) assays, respectively (Table). The two antibody pairs in the biplexed assay provide different LAM estimates due to the prevalence of specific epitopes in uLAM to which each antibody binds to, and the heterogeneous mixture of uLAM structures in urine.^5^ The amount of total protein and total carbohydrate were 14.35 mg and 258.7 μg, respectively. This filtered urine was the starting material for the enrichment and purification steps. The urine was concentrated using a Vivaflow 200 (10-kDa MWCO; Sartorius, Goettingen, Germany), and the retentate was washed in situ with ice-cold phosphate-buffered saline (Step 2). This step retained 83.0% (FA) and 71.0% (OA) uLAM and removed 93.4% of the total proteins and other interfering substances detected by the bicinchoninic assay and 96.4% of the carbohydrates. About 5.5% (FA) and 23.9% (OA) uLAM were lost in the flow-through. It is likely that LAM, with an average molecular weight of 17.4 kDa, was partially fragmented in urine in sizes small enough to pass through the 10-kDa filter. A slight loss of uLAM was also likely due to non-specific binding to the filter, or the uLAM being in complexes with other molecules bound to the filter. The reduction in total volume aided proteinase K digestion of the remaining protein. Proteinase K (Fisher Bioreagents, MA, USA) treatment followed by heat inactivation (Step 3), resulted in a significant increase in measured uLAM based on FA (but not OA) compared to the previous step. This is possibly the result of more epitopes for FA antibody pair being exposed after proteolysis.^14^ The increase in total protein concentration is due to the added proteinase K and from the digested peptides in the treated urine concentrate. Proteinase K and digested peptides were then removed using chloroform extraction (Step 4). This solvent extraction resulted in a ~30% loss of uLAM (despite backwashing the solvent phase) and was due to LAM being trapped in the insoluble aggregates in the interphase or the chloroform layer.

**Table:** Calculated amount of uLAM, protein and carbohydrate in each sequential processing steps

Step	Method*	uLAM^†‡^				Protein		Carbohydrate	

		FA		OA					
			
		ng (ng after ultrafiltration)	%	ng (ng after ultrafiltration)	%	μg (μgafter ultrafiltration)	%	μg (μgafter ultrafiltration)	%
1	Low-speed centrifugation and microfiltration	486.9	NA	102.8	NA	14,349.6	NA	258.7	NA
2	Ultrafiltration	403.9 (26.6)	83.0 (5.5)	73.1 (24.6)	71.0 (23.9)	69.9 (13,409.0)	0.5 (93.4)	8.0 (249.4)	3.1 (96.4)
3	Proteinase K treatment	463.6	95.2	74.1	72.1	76.9	0.5	8.9	3.5
4	Chloroform extraction	309.0	63.5	47.1	45.8	2.8	0.02	5.3	2.0
5, 6	Size exclusion chromatography, followed by ultrafiltration	250.4	51.4	32.1	31.2	2.3	0.02	1.3	0.5

* Detailed methodology in the Supplementary Data.

† The uLAM concentration was measured using a biplexed LAM immunoassay on a U-PLEX assay platform (Meso Scale Diagnostics, Rockville, MD, USA), with FIND28 (FIND) and S4-20 (Otsuka Pharmaceutical, Tokyo, Japan) as the capture antibodies and A194-01 (Rutgers University, NJ, USA) as the detector. The total amount of proteins and carbohydrates in each fraction was measured using the bicinchoninic acid and phenol-sulfuric acid methods, respectively. The amount and percentile relative to the starting amounts in Step 1 are shown, with quantities normalized per mL of material/resulting fractions from each step; in parenthesis are quantities in the filtrate after ultrafiltration.

‡ FA (FIND28/A194-01) and OA (Otsuka S4-20/A194-01) antibody pairs. uLAM = urine lipoarabinomannan; FA = FIND antibody; OA = Otsuka antibody; NA = not applicable.

This step effectively removed about 96% of protein (from 76.9 to 2.8 μg), with only 0.02% of the original amount of protein remaining. Size exclusion chromatography (SEC) using Sephacryl S-100HR column (Cytiva, MA, USA) further removed unwanted soluble materials from the chloroform-treated aqueous phase (Step 5). After concentrating the pooled uLAM-containing SEC fractions (Supplementary Figure S2) using a 10-kDa MWCO filter (Millipore-Sigma, MA, USA) (Step 6), the total carbohydrate was reduced further to 0.5% of the initial amount. Steps 5 and 6 had a negative effect on the recovery of uLAM, with losses of 19.0% and 31.8% when measured by FA and OA, respectively. Overall, 31% (OA) or 51% (FA) of uLAM was recovered and 99% of total protein and carbohydrate were removed from the contrived urine. Due to the limited amount of purified material, we were not able to demonstrate uLAM purity via gel electrophoresis and Western blotting, and compare it with culture-derived LAM.^7^

These combination of methods effectively enriched and purified uLAM, with up to 51% of uLAM recovered from the input amount. We deliberately excluded antibody-aided affinity chromatography in our approach to prevent the bias of enriching uLAM derivatives with epitopes that were already recognized by the currently available antibodies and conversely, losing uLAM with novel epitopes. The methods comprised a mixture of physical processes (e.g., centrifugation and filtration), combined with enzymatic and chemical treatments, using typical laboratory equipment. The approach is straightforward to scale up for high yields of purified uLAM. We envisage that by offering a simple purification method, urine samples with grade 3–4 scores from the Determine TB LAM assay (Abbott, IL, USA) can be collected and pooled into large volumes, and then processed to yield sufficient amounts of uLAM for further characterization and analysis. This purified material would be a valuable tool for LAM antibody generation, from selecting antibody-producing B-cells to screening new and better performing uLAM-specific antibodies. Furthermore, more suitable uLAM material can support the early development and performance verification of new or existing TB LAM diagnostic assays in lieu of purified LAM from in vitro cultured MTB. This can inform if such assays can meet the required clinical performance ranges as specified in the target product profiles for either a triage tool or an RDT for TB.^15^
